# The safety of vedolizumab in a patient with Crohn’s disease who developed anti-TNF-alpha agent associated latent tuberculosis infection reactivation: A case report

**DOI:** 10.1097/MD.0000000000034331

**Published:** 2023-07-14

**Authors:** Yuya Sugiyama, Nobuhiro Ueno, Shion Tachibana, Yu Kobayashi, Yuki Murakami, Takahiro Sasaki, Aki Sakatani, Keitaro Takahashi, Katsuyoshi Ando, Shin Kashima, Kentaro Moriichi, Hiroki Tanabe, Toshikatsu Okumura, Mikihiro Fujiya

**Affiliations:** a Division of Metabolism and Biosystemic Science, Gastroenterology, and Hematology/Oncology, Department of Medicine, Asahikawa Medical University, Asahikawa, Japan; b Division of General Medicine, Asahikawa Medical University Hospital, Asahikawa, Japan.

**Keywords:** active tuberculosis infection, Crohn’s disease, latent tuberculosis infection, *Mycobacterium tuberculosis*, Vedolizumab

## Abstract

Latent tuberculosis (TB) infection screening before inducing anti-tumor necrosis factor (anti-TNF) alpha agents is important to prevent TB reactivation. However, latent TB infection reactivation may still occur, and the ideal therapeutic strategy for patients with inflammatory bowel disease (IBD) who develop active TB infection has not been established. Vedolizumab (VDZ) has a good safety profile, with low incidence rates of serious infections. However, its safety in patients with latent TB infection reactivation associated with anti-TNF-alpha agents remains unknown.

**Patient concerns:**

A 21-year-old Vietnamese male patient presented to our hospital with hemorrhagic stool. He had no personal or family history of IBD or TB.

**Diagnoses:**

Colonoscopy revealed multiple longitudinal ulcers and a cobblestone appearance in the terminal ileum, as well as multiple small erosions and aphtha throughout the colon. Computed tomography revealed a right lung nodular lesion. Serological interferon-gamma release assay and several culture tests were all negative. Thus, he was diagnosed with ileocolonic Crohn’s disease (CD) without TB.

**Interventions:**

The intravenous anti-TNF-alpha agent administration with an immunomodulator was initiated.

**Outcomes:**

Computed tomography revealed nodular lesion expansion at the right lung, and serological interferon-gamma release assay was positive. He was diagnosed with latent TB infection reactivation. Anti-TNF-alpha agent with an immunomodulator was immediately discontinued, and anti-TB therapy was initiated. His endoscopic findings were still active, and VDZ was selected for maintenance therapy because VDZ has a favorable safety profile with low incidence rates of serious infections. Consequently, mucosal healing was achieved without active TB relapse.

**Lessons:**

This case report presented a patient in whom VDZ was continued as maintenance therapy without inducing TB relapse in a patient with CD who developed latent TB infection reactivation associated with anti-TNF-alpha agents and summarized the safety profile of VDZ for patients with IBD with active or latent TB infection. VDZ may be a safe option for induction and maintenance therapy in patients with CD, even in cases with latent TB infection reactivation.

## 1. Introduction

Anti-tumor necrosis factor (anti-TNF) alpha agents have a high therapeutic efficacy and safety profile for inflammatory bowel disease (IBD), and a small percentage of patients experience severe adverse effects, including infectious disease.^[1]^ TNF-alpha plays an important role in the host defense against *Mycobacterium tuberculosis*, thus anti-TNF-alpha agents are associated with an increased risk of active tuberculosis (TB).^[2]^ Active TB infection is mostly developed within several months after anti-TNF alpha agent initiation, suggesting latent TB infection reactivation as their cause.^[3]^ Latent TB infection screening before anti-TNF-alpha agent induction is important for preventing TB reactivation. Many scientific organizations have published guidelines and recommendations concerning active and latent TB infection.^[4–7]^ However, latent TB infection reactivation still occurs in patients with IBD treated with anti-TNF-alpha agents despite compliance with recommended preventive measures.^[8]^ Furthermore, therapeutic strategies for patients with IBD who develop anti-TNF-alpha agent-associated active TB infection have not yet been established.

Vedolizumab (VDZ), which is a gut-selective antibody to α4β7 integrin for IBD treatment, has a favorable safety profile with low incidence rates of serious infections, infusion-related reactions, and malignancies.^[9]^ However, its safety for patients with anti-TNF-alpha agent-associated active TB infection remains unclear.

Herein, we report the successful continuation of VDZ as maintenance therapy without TB relapse induction in a patient with Crohn’s disease (CD) who developed anti-TNF-alpha agent-associated latent TB infection reactivation.

## 2. Case presentation

**Figure 1.**
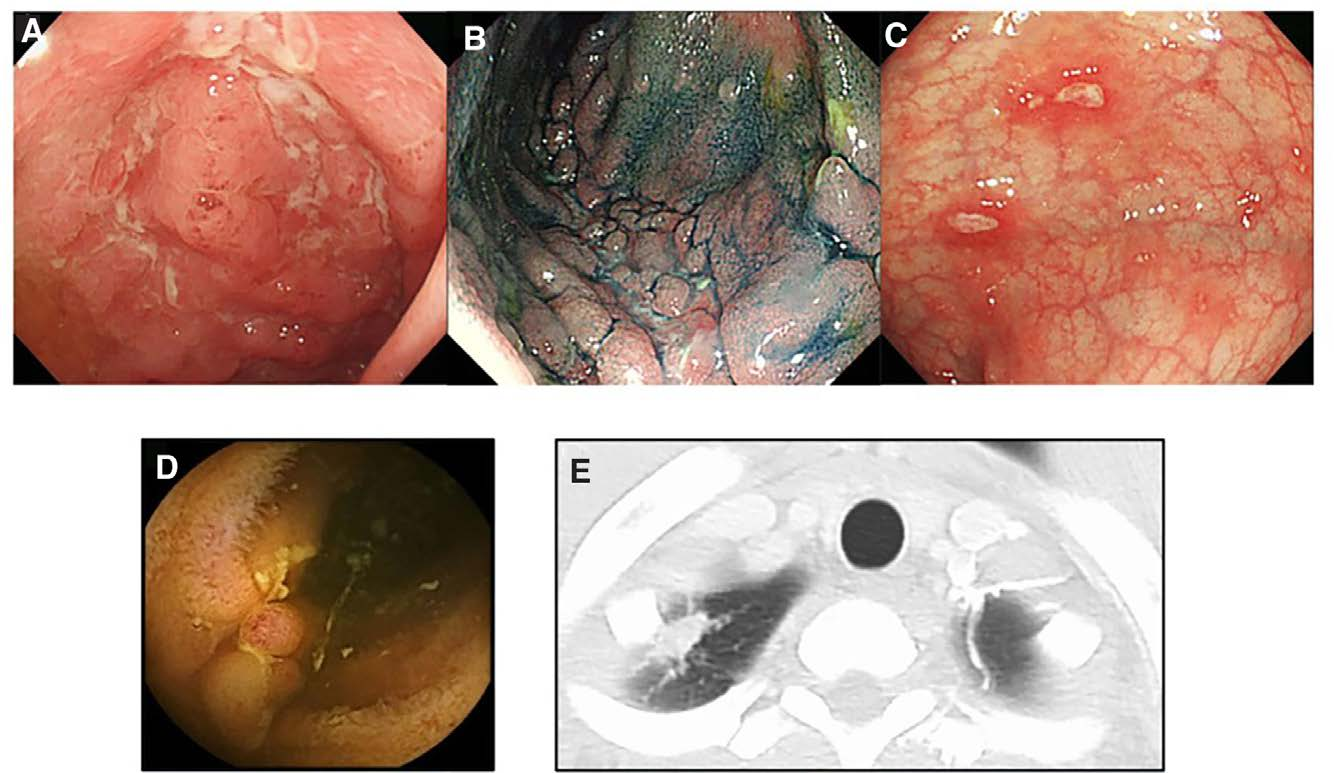
(A–C) Colonoscopic findings: multiple longitudinal ulcers and a cobblestone appearance in the terminal ileum and multiple small aphthae in the colon at the initial diagnosis. (D) Small bowel capsule endoscopic findings: a cobblestone appeared in the ileum at the initial diagnosis. (E) Chest computed tomography findings: a nodular lesion at the apex of the right lung before the administration of anti-TNF-alpha agent.

A 21-year-old Vietnamese male patient presented to Asahikawa Medical University Hospital with hemorrhagic stool. He had no personal or family history of IBD or TB. A physical examination revealed the following: body height of 171.0 cm; body weight of 63.8 kg; body mass index of 21.8 kg/m^2^; body temperature of 36.7°C; blood pressure of 115/58 mm Hg; heart rate of 71 beats/min; and peripheral capillary oxygen saturation of 99% on room air. A laboratory examination revealed high inflammation marker levels, mild anemia, and hypoalbuminemia, with white blood cell (WBC) count of 11,060/µL, hemoglobin of 12.9 g/dL, platelet count of 33.3 × 10^4^/µL, C-reactive protein (CRP) of 4.43 mg/dL, and albumin of 3.7 g/dL. Serological tests for infection markers, including the interferon-gamma release assay (IGRA), were all negative. Upper gastrointestinal endoscopy revealed multiple vertical small erosions in the duodenum. Colonoscopy revealed multiple longitudinal ulcers and a cobblestone appearance in the terminal ileum and multiple small erosions and aphtha throughout the colon (Fig. 1A–C). Findings from intestinal juice culture examination and polymerase chain reaction for any bacteria, including acid-fast bacilli, were negative. Small bowel capsule endoscopy revealed multiple small ulcers in the jejunum and multiple longitudinal ulcers and a cobblestone appearance in the ileum (Fig. 1D). Contrast-enhanced computed tomography (CT) revealed a nodular lesion at the apex of the right lung as well as wall thickening with a contrast effect at the terminal ileum (Fig. 1E). A pathological examination revealed no granuloma and acid-fast bacilli at the longitudinal ulcer in the terminal ileum.

He was diagnosed with ileocolonic CD following the endoscopic findings. The nodular lesion at the apex of the right lung was suspected to be latent TB. However, his serological IGRA and several culture tests (including sputum, urine, and intestinal juice) were all negative. Thus, he was not diagnosed with a latent TB infection. CD activity had a Crohn’s disease activity index (CDAI) of 167, Lewis score of 1368, and simple endoscopic score for Crohnʼs disease (SES-CD) of 13. The intravenous anti-TNF-alpha agent administration (5 mg/kg) with an immunomodulator (50 mg/body) was initiated as induction therapy because of his young age and the presence of high-activity inflammatory lesions throughout the small intestine and colon.

**Figure 2.**
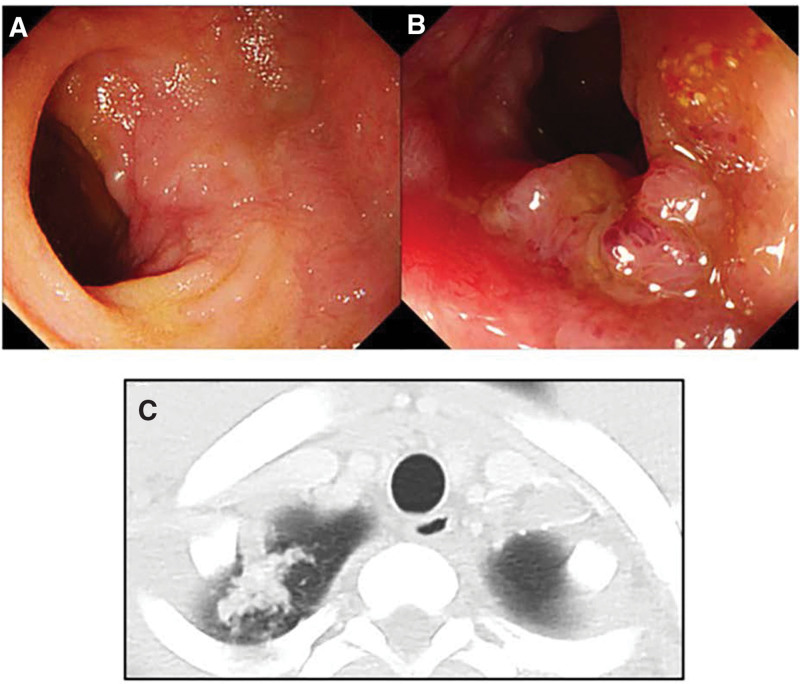
(A and B) Colonoscopy findings: improved lesions and a remained active ulcer in the terminal ileum. (C) Chest computed tomography findings: a worsening nodular lesion 17 wk after the anti-TNF-alpha agent initiation.

His CDAI and CRP levels immediately decreased after anti-TNF-alpha agent induction. However, he visited our hospital with a high fever 17 weeks after the anti-TNF-alpha agent initiation. A laboratory examination revealed high inflammation markers levels, with a WBC count of 6560/µL and CRP of 10.33 mg/dL. CT revealed the nodular lesion expansion at the apex of the right lung (Fig. 2C). Furthermore, both his serological IGRA, and acid-fast bacillus culture tests of gastric juice became positive. He was diagnosed with latent TB infection reactivation. Anti-TNF-alpha agent with an immunomodulator was immediately discontinued, and anti-TB therapy with rifampicin, isoniazid, ethambutol, and pyrazinamide was initiated. His general condition and inflammation markers had recovered 10 weeks after the anti-TB therapy initiation, with a WBC count of 4850/µL and CRP of <0.10 mg/dL.

The patient maintained clinical remission of CD without medication in July 2019. Colonoscopy revealed improved lesions but an active ulcer remained in the terminal ileum (Fig. 2A and B). He had a CDAI of 85 and an SES-CD of 4. His endoscopic findings were improved compared with those before anti-TNF-alpha agent induction therapy, but mucosal healing was not achieved thus, another intervention therapy for CD that would not induce a relapse of active TB was considered. VDZ (300 mg/body) was selected for maintenance therapy because VDZ has a favorable safety profile with low incidence rates of serious infections.

**Figure 3.**
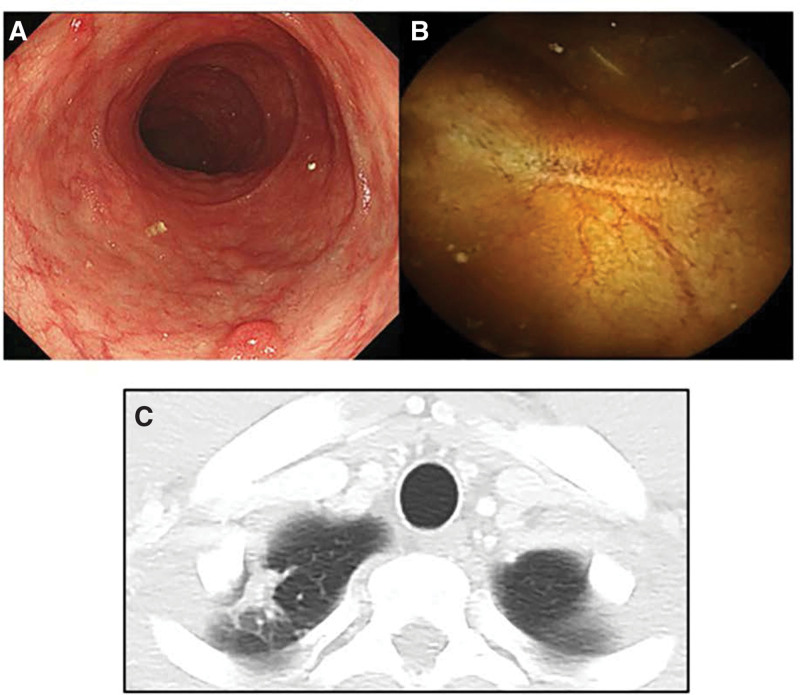
(A) Colonoscopy findings: mucosal healing in the terminal ileum 1 yr after VDZ administration. (B) Small bowel capsule endoscopic findings: mucosal healing in the whole small intestine 1 yr after VDZ administration. (C) Chest computed tomography findings: a shrunken nodular lesion 1 yr after VDZ administration. VDZ = vedolizumab.

All his ulcerative lesions in the small intestine and colon had improved and changed to ulcer scars on small bowel capsule endoscopy and colonoscopy in June 2020 (Fig. 3A and B). He had a CDAI of 80, Lewis score of 0, and SES-CD of 0. CT revealed shrinkage of the nodular lesion at the apex of the right lung (Fig. 3C). Thus, he achieved both clinical remission and mucosal healing with VDZ, as maintenance therapy, without active TB infection relapse.

## 3. Discussion

This case report showed continued VDZ as maintenance therapy without TB relapse in a patient with CD who developed anti-TNF-alpha agent-associated latent TB infection reactivation. VDZ is considered a safe and effective therapy for patients with CD who develop latent TB infection reactivation.

TNF-alpha plays a central role in the host’s immune defense against TB infection. Therefore, inhibiting TNF-alpha may increase TB susceptibility and accelerate latent TB infection reactivation.^[2]^ The prevalence of latent TB infection is much higher in the general population of Asian countries than in Western countries. The European Crohn’s and Colitis Organization, the British Society of Gastroenterology, and the Asian Organization for Crohn’s and Colitis recommend that patients with IBD should be screened for TB infection by chest radiography and a tuberculin skin test and/or IGRA before anti-TNF agent initiation.^[4–7]^ CT revealed a nodular lesion at the apex of the right lung in the present case. However, he did not have any symptoms, and his IGRA, and several culture tests as well as a polymerase chain reaction examination were all negative. Therefore, he was not diagnosed with a latent TB infection. Lu et al^[10]^ reported that IGRA demonstrated a superior capability to the tuberculin skin test for diagnosing TB because of its markedly greater sensitivity and specificity. However, Santos et al^[11]^ reported that host factors, including inflammatory disease and pulmonary TB, are associated with false-negative IGRA results. Thus, the ideal way to diagnose latent TB infection has not yet been established. Patients with IBD having pulmonary lesions on CT should receive chemoprophylaxis before the anti-TNF agent initiation even if IGRA is negative, as in our case.

The European Crohn’s and Colitis Organization and Asian Organization for Crohn’s and Colitis suggest that anti-TNF-alpha agents were withheld when active TB infection is diagnosed during the drug administration.^[4,6,7]^ However, the British guideline contrarily recommends that anti-TNF-alpha agents should be continued if clinically indicated.^[5]^ Thus, there is little evidence of the risk of active TB infection by immunosuppressive therapies, including anti-TNF-alpha agents. Active lesions in the small intestine remained when the anti-TNF agent with an immunomodulator was discontinued in the present case. Achieving mucosal healing in patients with CD is generally associated with improved rates of long-term clinical remission.^[12]^ Thus, we selected another therapeutic option, VDZ, which was thought to have little effect on TB reactivation.

VDZ is a humanized monoclonal antibody that selectively binds to the α_4_β_7_ integrin. VDZ inhibits lymphocyte adhesion to mucosal addressin cell adhesion molecule-1 and subsequent migration. The safety of VDZ for patients with IBD with active or latent TB infection was summarized in Table 1. Several clinical trials and meta-analyses have demonstrated a low risk of developing latent TB infection reactivation with VDZ.^[9,13–16]^ A prospective observation study revealed that only 1 out of 294 patients had developed active TB infection despite a negative pretherapeutic screening.^[17]^ Furthermore, a retrospective study of patients with IBD with latent TB infection who received VDZ revealed no cases of active TB infection.^[18–20]^ Choi et al reported a case of a pediatric patient with CD who successfully achieved and maintained remission with VDZ after developing pulmonary TB during infliximab treatment. This case report is very similar to the present case. Our patient also achieved both clinical remission and mucosal healing by VDZ maintenance therapy without an active TB infection relapse. Thus, VDZ is expected to be a safe treatment even in patients who develop active TB infection based on the present case and the previous reports describing the extremely low risk of latent TB infection reactivation.

**Table 1:** The safety of VDZ for the IBD patient with active or latent TB infection.

Publication	Type of study	Country	Patients	Key findings
Colombel et al (2017)^[9]^	Integrated analysis (GEMINI 1, GEMINI 2, GEMINI 3, and GEMINI LTS)	N/A	2,932	Four patients developed TB infection (0.13%). Three were considered primary infection, and 1 developed latent TB infection
Loftus et al (2020)^[13]^	Phase 3, single-arm, open-label, multinational study (GEMINI LTS)	N/A	2,243	Four patients developed TB (0.17%). Three were considered primary infection
Ng et al (2018)^[14]^	Integrated analysis (GEMINI 1, GEMINI 2, GEMINI LTS, and post-marketing AE reports)	N/A	N/A	Tuberculosis was reported at 0.1 per 100 patient-years (clinical trials), with 7 events in the post-marketing setting
Cohen et al (2020)^[15]^	Retrospective study of post-marketing AE reports	N/A	32,752	Nine patient developed TB infection (0.03%). Three developed latent TB infection
Bonovas et al (2016)^[16]^	Meta-analysis	N/A	461	Odds ratio of TB numerically higher with VDZ vs placebo (2.50, 95% CT, 0.06–104.43). Result did not reach statistical significance
Amiot et al (2016)^[17]^	Prospective observation study	France	294	One patient developed TB infection (0.34%) despite a negative pretherapeutic screening
Ramos et al (2018)^[18]^	Retrospective study of IBD patients who received biologic with LTBI	USA	7	None developed active TB infection (0%) in 7 patients treated with VDZ
Kim et al (2021)^[19]^	Retrospective study of IBD patients who developed LTBI	Korea	47	Positive IGRA conversion was observed in 4 patients (8.5%), none of the 4 converters developed active TB infection
Choi et al (2022)^[20]^	Retrospective study (real world data)	Korea	125	None developed active TB infection (0%) in patients treated with VDZ
Choi et al (2021)^[21]^	Case report	Korea	1	A case of a pediatric CD patient who successfully achieved and maintained remission with VDZ after developing pulmonary TB during treatment with IFX

AE = adverse events, CD = Crohn’s disease, IBD = inflammatory bowel disease, IFX = infliximab, IGRA = interferon-gamma release assay, TB = tuberculosis, VDZ = vedolizumab.

In conclusion, this case report described a patient with CD who developed anti-TNF-alpha agent-associated latent TB infection reactivation in which VDZ was continued as maintenance therapy without inducing TB relapse and summarized the safety profile of VDZ for patients with IBD with active or latent TB infection. Clinicians must be alert for latent TB infection reactivation during anti-TNF-alpha agent administration, particularly in Asian countries. VDZ may be a safe option for therapy induction and maintenance in patients with CD, even in cases with latent TB infection reactivation associated with anti-TNF-alpha agents. A new therapeutic strategy using VDZ for patients with CD with TB infection is expected to be established following the accumulation of similar cases.

## Author contributions

**Conceptualization:** Nobuhiro Ueno, Yuya Sugiyama.

**Data curation:** Yuya Sugiyama, Shion Tachibana, Yu Kobayashi, Yuki Murakami, Takahiro Sasaki, Keitaro Takahashi, Katsuyoshi Ando, Shin Kashima, Kentaro Moriichi, Hiroki Tanabe.

**Methodology:** Nobuhiro Ueno, Aki Sakatani, Mikihiro Fujiya.

**Validation:** Nobuhiro Ueno, Katsuyoshi Ando, Mikihiro Fujiya.

**Writing – original draft:** Nobuhiro Ueno, Yuya Sugiyama.

**Writing – review & editing:** Nobuhiro Ueno, Toshikatsu Okumura, Mikihiro Fujiya.
